# Runx2 in the Perichondrial Osteoblasts Enhances Terminal Differentiation of Chondrocytes Through *Nell1* Induction

**DOI:** 10.3390/ijms27031266

**Published:** 2026-01-27

**Authors:** Xin Qin, Qing Jiang, Longfei Wu, Suemi Yabuta, Chiharu Sakane, Yuki Matsuo, Ziheng Zhang, Hisato Komori, Manyu Zhang, Kosei Ito, Toshihisa Komori

**Affiliations:** 1 Institute of Orthopaedics, Suzhou Medical College, Soochow University, Suzhou 215006, China; xqin@suda.edu.cn (X.Q.); qjiang1006@suda.edu.cn (Q.J.);; 2 Center for Genetic Epidemiology and Genomics, School of Public Health, Suzhou Medical College, Soochow University, Suzhou 215006, China; 3 Department of Molecular Tumor Biology, Nagasaki University Graduate School of Biomedical Sciences, Nagasaki 852-8588, Japan; 4 Research Center for Biomedical Models and Animal Welfare, Nagasaki University Graduate School of Biomedical Sciences, Nagasaki 852-8588, Japan; 5 Department of Skeletal Development and Regenerative Biology, Nagasaki University Graduate School of Biomedical Sciences, Nagasaki 852-8588, Japan

**Keywords:** Runx2, perichondrium, chondrocyte differentiation, Nell1, endochondral ossification, terminal hypertrophic chondrocytes

## Abstract

Runx2 plays essential roles in osteoblast differentiation and chondrocyte maturation. Runx2 in the perichondrium has been reported to inhibit chondrocyte maturation through *Fgf18* induction. To further investigate the functions of Runx2 in the perichondrium, we generated *Runx2*^fl/−Cre^ mice by crossing *Runx2*^fl/+^, *Runx2*^+/−^, and 2.3-kb *Col1a1* Cre mice and compared them with *Runx2*^fl/−^ mice at E15.5, when the endochondral bones were cartilaginous. Skeletal preparation of the upper limbs in *Runx2*^fl/−Cre^ mice showed reduced mineralization of the humerus and scapula, and histological analysis of the femurs showed delays in the terminal differentiation of chondrocytes, as indicated by the absence of mineralization and *Spp1* expression in the cartilage and osteoblast differentiation in the perichondrium, compared to those in *Runx2*^fl/−^ mice. mRNA sequence analysis showed that the expression of *Nell1*, which encodes a secreted protein that enhances chondrocyte maturation, in *Runx2*^fl/−Cre^ femurs was more than two-fold lower than that in *Runx2*^fl/−^ femurs. Nell1 expression was reduced in the perichondrium of *Runx2*^fl/−Cre^ femurs compared to that in *Runx2*^fl/−^ femurs. *Nell1* expression was upregulated by *Runx2* overexpression and downregulated by *Runx2* siRNA. These findings indicate that Runx2 in perichondrial osteoblasts enhances the terminal differentiation of chondrocytes by inducing *Nell1* expression.

## 1. Introduction

During endochondral ossification, mesenchymal cells condense and differentiate into chondrocytes, forming cartilaginous skeletons. Chondrocytes proliferate and mature into prehypertrophic, hypertrophic, and terminal hypertrophic chondrocytes. Osteoblast differentiation occurs in the perichondrium surrounding the prehypertrophic, hypertrophic, and terminal hypertrophic chondrocyte layers, forming bone collars. Furthermore, the matrix of the terminal hypertrophic chondrocyte layer is mineralized [1,2]. Runx2 is upregulated in prehypertrophic chondrocytes and induces chondrocyte maturation. Upregulated Runx2 expression is maintained in hypertrophic and terminal hypertrophic chondrocytes. Runx2 induces *Ihh* expression in prehypertrophic chondrocytes, Ihh induces *Pthlh* expression in the periarticular growth plate, and Pthlh inhibits chondrocyte maturation by inhibiting *Runx2* expression in prehypertrophic chondrocytes through Pth1r, forming a negative feedback loop [3,4,5]. Ihh also induces *Runx2* expression and osteoblast differentiation in the perichondrium [5,6,7]. Therefore, chondrocyte maturation induces osteoblast differentiation in the perichondrium.

The perichondrium was shown to inhibit chondrocyte maturation. In the organ culture of chicken tibiotarsi, in which the perichondrium was removed, chondrocyte maturation was accelerated, indicating that the perichondrium inhibits chondrocyte maturation [8]. Furthermore, it has been reported that Runx2 in the perichondrium inhibits chondrocyte maturation by inducing *Fgf18* expression [9]. In *Fgf18*^−/−^ mice, chondrocyte proliferation in the resting and proliferating layers of the growth plate was increased, and the proliferating, prehypertrophic, and hypertrophic chondrocyte layers were elongated; however, the differentiation of hypertrophic chondrocytes into terminal hypertrophic chondrocytes was inhibited, as shown by the reduction in *Spp1*-expressing terminal hypertrophic chondrocytes, resulting in the retardation of endochondral ossification [10,11]. Although the enlarged *Col10a1*-positive hypertrophic chondrocyte layer in *Fgf18*^−/−^ mice was considered to be caused by enhanced chondrocyte maturation [10,11], it was likely due to the enhanced proliferation and inhibited terminal differentiation of chondrocytes. Thus, Fgf18 inhibits chondrocyte proliferation but is not likely to inhibit chondrocyte maturation and enhances terminal differentiation of chondrocytes. Twist1, which is expressed in the perichondrium, interacts with Runx2 and inhibits Runx2 function, and *Twist1* mutant mice have shown a reduced *Col10a1*-positive hypertrophic chondrocyte layer due to decelerated chondrocyte maturation [9]. However, the phenotypes showed that the reduced *Col10a1*-positive hypertrophic chondrocyte layer was caused by enhanced chondrocyte maturation and endochondral ossification. Thus, the misinterpretation of the phenotypes of *Fgf18*^−/−^ and *Twist1* mutant mice makes it difficult to evaluate Runx2 function in the perichondrium during chondrocyte maturation.

Nel-like molecule-1 (Nell1), a neuroepidermal growth factor-like secreted protein, has been identified as an upregulated gene in coronal synostosis [12]. Overexpression of *Nell1* causes craniosynostosis, and *Nell1* mutant mice exhibit skeletal defects in the skull, vertebral column, and rib cage [13,14]. Nell1 is expressed in osteoblasts, chondrocytes, and the perichondrium and induces the differentiation of osteoblasts and chondrocytes [13,15,16,17,18,19]. Chondrocyte maturation was inhibited in *Nell1* mutants and chondrocyte-specific *Nell1* deficient mice, and Nell1 induced chondrocyte maturation [16,17,18]. Moreover, Runx2 directly regulates the *Nell1* promoter, cranial defects in *Runx2*^+/−^ mice were partially rescued by crossing with *Nell1* overexpression transgenic mice or adding recombinant Nell1 in the organ culture, and *Nell1* expression is regulated by Runx2 in chondrocytes [16,19,20].

We previously reported that Runx2 is required for the expression of major bone matrix protein genes in osteoblasts by generating osteoblast-specific *Runx2*-deficient mice using 2.3 kb *Col1a1*-enhanced green fluorescent protein (EGFP)-Cre mice (*Runx2*^fl/flCre^ mice) [21]. To investigate Runx2 functions in the perichondrium, we generated *Runx2*^fl/−Cre^ mice and analyzed them at E15.5, when the endochondral bones were cartilaginous. We found that deletion of *Runx2* in perichondrial osteoblasts decelerated the terminal differentiation of chondrocytes. Therefore, contrary to a previous report [9], Runx2 in the perichondrium enhances chondrocyte maturation. We investigated the mechanism of the regulation of chondrocyte maturation by Runx2 in the perichondrium and found that Nell1 expression in the perichondrium of *Runx2*^fl/−Cre^ mice was reduced compared with that of *Runx2*^fl/−^ mice at E15.5. Overexpression of *Runx2* induced *Nell1* expression and knockdown of *Runx2* reduced it, indicating that Runx2 in perichondrial osteoblasts enhances the terminal differentiation of chondrocytes, at least in part, through the induction of *Nell1* expression.

## 2. Results

### 2.1. Mineralization of Ribs, Vertebrae, Clavicles, Humeri, and Scapulae in Runx2^fl/−Cre^ Mice Was Less than That in Runx2^fl/−^ Mice at E15.5

**Figure 1:**
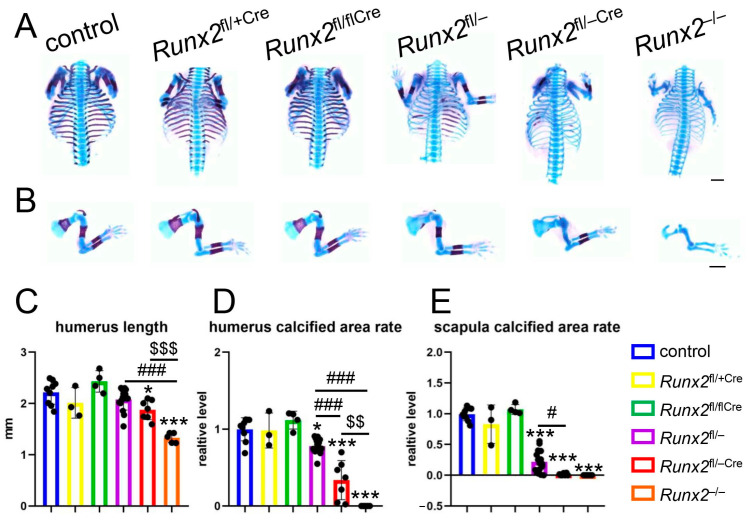
Skeletal systems of control (*Runx2*^fl/+^ and *Runx2*^fl/fl^), *Runx2*^fl/+Cre^, *Runx2*^fl/flCre^, *Runx2*^fl/−^, *Runx2*^fl/−Cre^, and *Runx2*^−/−^ embryos at E15.5. (**A**) Frontal view of the upper body skeletons. (**B**) Lateral view of the upper limbs. Scale bars = 1 mm. (**C**) Length of humerus. (**D**) Ratio of calcified area in the humerus. (**E**) Ratio of the calcified area in the scapula. The number of mice analyzed: control: 9, *Runx2*^fl/+Cre^: 3, *Runx2*^fl/flCre^: 4, *Runx2*^fl/−^: 16, *Runx2*^fl/−Cre^: 7, *Runx2*^−/−^: 5. Data are the mean ± SD. * Versus control mice, # versus *Runx2*^fl/−^ mice, $ versus *Runx2*^fl/−Cre^ mice. *, # *p* < 0.05, $$ *p* < 0.01, ***, ###, $$$ *p* < 0.001.

We generated *Runx2*^fl/−Cre^ mice by crossing *Runx2*^fl/+^, 2.3 kb *Col1a1* EGFP-Cre, and *Runx2*^+/−^ mice. We compared the skeletal preparation of control (*Runx2*^fl/+^ and *Runx2*^fl/fl^), *Runx2*^fl/+Cre^, *Runx2*^fl/flCre^, *Runx2*^fl/−^ (or *Runx2*^+/−^), *Runx2*^fl/−Cre^, and *Runx2*^−/−^ mice. The mineralization of the ribs, vertebrae, clavicles, humeri, and scapulae was similar among the control, *Runx2*^fl/+Cre^, and *Runx2*^fl/flCre^ mice (Figure 1A,B,D,E). Histological analysis of the femurs also showed that the lengths of the femurs and the resting, proliferating, and hypertrophic chondrocyte layers were similar between *Runx2*^fl/fl^ and *Runx2*^fl/flCre^ mice (Supplementary Figure S1). Compared with the control, *Runx2*^fl/+Cre^, and *Runx2*^fl/flCre^ mice, the mineralization of the ribs, vertebrae, clavicles, humeri, and scapulae was reduced in *Runx2*^fl/−^ mice, further reduced in *Runx2*^fl/−Cre^ mice, and absent in *Runx2*^−/−^ mice (Figure 1A,B,D,E). The length of the humeri in *Runx2*^fl/−Cre^ mice but not *Runx2*^fl/−^ mice was shorter than that in control mice and further reduced in *Runx2*^−/−^ mice (Figure 1C).

### 2.2. The Process of Endochondral Ossification Was Retarded in Runx2^fl/−Cre^ Mice Compared with That in Runx2^fl/fl^ and Runx2^fl/flCre^ Mice

**Figure 2:**
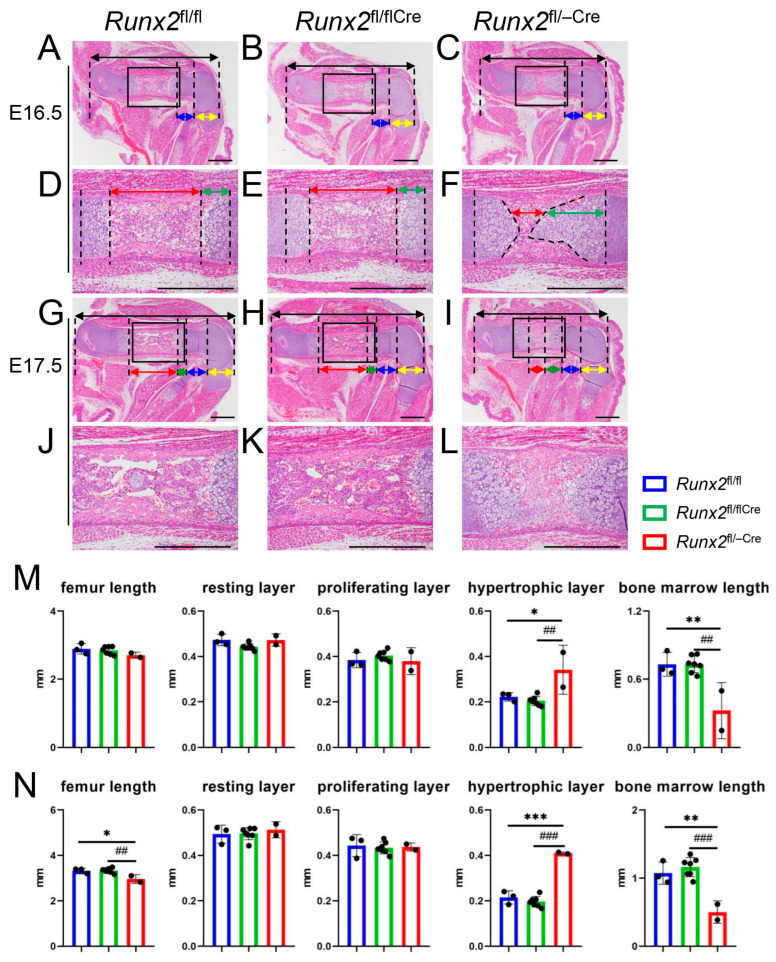
Histological analyses of *Runx2*^fl/fl^, *Runx2*^fl/flCre^, and *Runx2*^fl/−Cre^ embryos at E16.5 and E17.5. (**A**–**L**) H&E staining of femoral sections at E16.5 (**A**–**F**) and E17.5 (**G**–**L**). (**A**,**D**,**G**,**J**) *Runx2*^fl/fl^, (**B**,**E**,**H**,**K**) *Runx2*^fl/−^, and (**C**,**F**,**I**,**L**) *Runx2*^fl/−Cre^ embryos. The boxed regions in (**A**–**C**) and (**G**–**I**) are magnified in (**D**–**F**) and (**J**–**L**), respectively. The lengths of the femur (black arrows), resting chondrocyte layer (yellow arrows), proliferating chondrocyte layer (blue arrows), hypertrophic chondrocyte layer (green arrows), and bone marrow (red arrows) are shown. Scale bars = 0.5 mm. (**M**) Lengths of the femurs, layers of resting, proliferating, and hypertrophic chondrocytes, and bone marrow at E16.5. (**N**) Same parameters at E17.5. The number of mice analyzed was as follows: *Runx2*^fl/fl^: 3, *Runx2*^fl/flCre^: 7, and *Runx2*^fl/−Cre^: 2, at E16.5 and E17.5. Data are the mean ± SD. * Versus *Runx2*^fl/fl^ mice, # versus *Runx2*^fl/−^ mice. * *p* < 0.05, **, ## *p* < 0.01, ***, ### *p* < 0.001.

Femurs were histologically analyzed at E16.5 and E17.5 (Figure 2). The femur lengths were comparable in *Runx2*^fl/fl^, *Runx2*^fl/flCre^, and *Runx2*^fl/−Cre^ mice at E16.5 (Figure 2A–C,M), whereas those in *Runx2*^fl/−Cre^ mice were shorter than those in *Runx2*^fl/fl^ and *Runx2*^fl/flCre^ mice at E17.5 (Figure 2G–I,N). The lengths of the resting and proliferating chondrocyte layers were comparable in *Runx2*^fl/fl^_,_ *Runx2*^fl/flCre^, and *Runx2*^fl/−Cre^ mice at E16.5 and E17.5, whereas those of the hypertrophic chondrocyte layer in *Runx2*^fl/−Cre^ mice were longer than those in *Runx2*^fl/fl^ and *Runx2*^fl/flCre^ mice (Figure 2A–I,M,N). Furthermore, the length of the bone marrow was severely reduced in *Runx2*^fl/−Cre^ mice compared to that in *Runx2*^fl/fl^ and *Runx2*^fl/flCre^ mice at E16.5 and E17.5 (Figure 2D–L,M,N), indicating that endochondral ossification was retarded in *Runx2*^fl/−Cre^ mice compared to *Runx2*^fl/fl^ and *Runx2*^fl/flCre^ mice.

### 2.3. Terminal Differentiation of Chondrocytes Was Retarded in Runx2^fl/−Cre^ Mice Compared with Runx2^fl/−^ Mice

**Figure 3:**
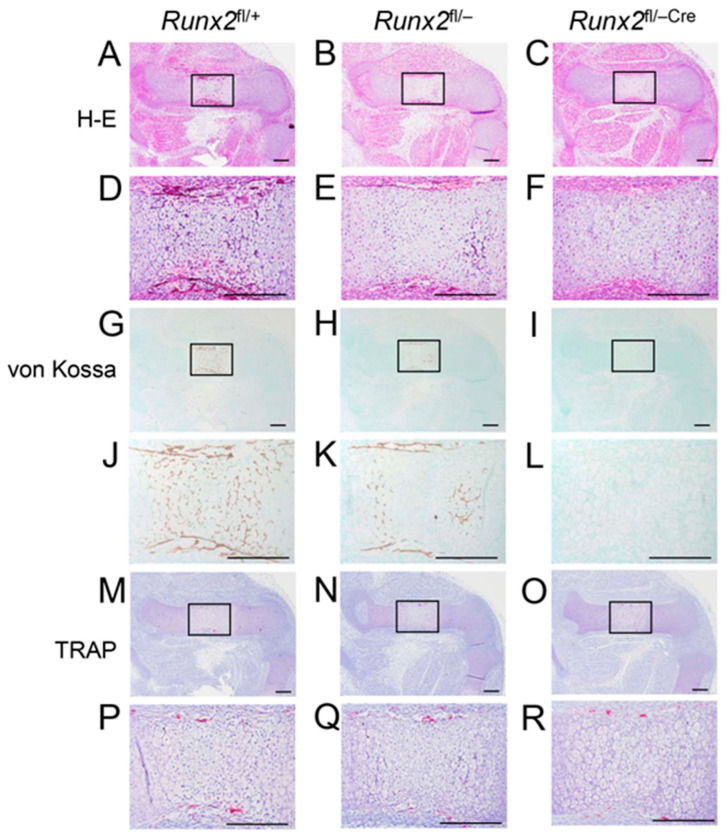
Histological analyses of femurs in *Runx2*^fl/+^, *Runx2*^fl/−^, and *Runx2*^fl/−Cre^ embryos at E15.5. (**A**–**F**) H&E staining. (**G**–**L**) Von Kossa staining. (**M**–**R**) TRAP staining. (**A**,**D**,**G**,**J**,**M**,**P**) *Runx2*^fl/+^, (**B**,**E**,**H**,**K**,**N**,**Q**) *Runx2*^fl/−^, and (**C**,**F**,**I**,**L**,**O**,**R**) *Runx2*^fl/−Cre^ embryos. The boxed regions in (**A**–**C**), (**G**–**I**), and (**M**–**O**) are magnified in (**D**–**F**), (**J**–**L**), and (**P**–**R**), respectively. Scale bars = 200 μm. The number of mice analyzed was as follows: *Runx2*^fl/+^: 4, *Runx2*^fl/flCre^: 5 and *Runx2*^fl/−Cre^: 4 in H&E staining; *Runx2*^fl/+^: 2, *Runx2*^fl/flCre^: 2 and *Runx2*^fl/−Cre^: 3 in von Kossa and TRAP staining.

Chondrocyte maturation and bone collar formation were examined in the femurs at E15.5 (Figure 3). Chondrocyte hypertrophy, but no vascular invasion, occurred at the diaphysis in *Runx2*^fl/+^, *Runx2*^fl/−^, and *Runx2*^fl/−Cre^ mice (Figure 3A–F). However, mineralization was observed in the diaphysis and bone collar in *Runx2*^fl/+^ and *Runx2*^fl/−^ mice but not in *Runx2*^fl/−Cre^ mice (Figure 3G–L), suggesting that terminal differentiation of chondrocytes and bone collar formation were retarded in *Runx2*^fl/−Cre^ mice. TRAP-positive cells were observed in the perichondrium of *Runx2*^fl/+^, *Runx2*^fl/−^, and *Runx2*^fl/−Cre^ mice (Figure 3M–R).

**Figure 4:**
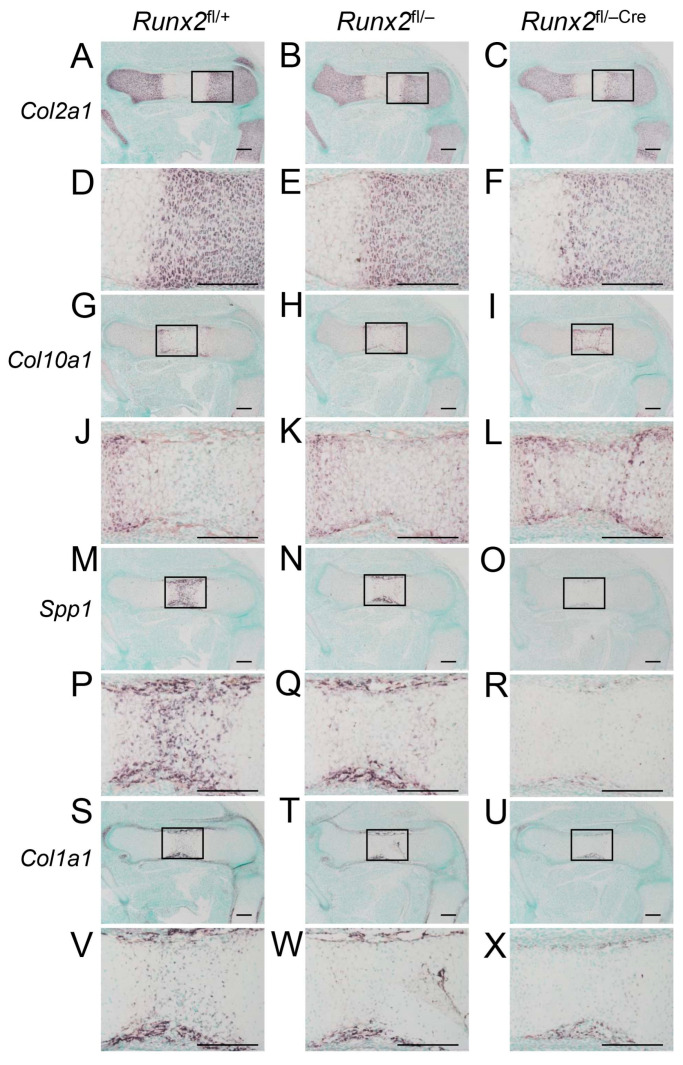
In situ hybridization of *Col2a1*, *Col10a1*, *Spp1*, and *Col1a1* at E15.5. (**A**–**X**) In situ hybridization of the femoral sections using *Col2a1* (**A**–**F**), *Col10a1* (**G**–**L**), *Spp1* (**M**–**R**), and *Col1a1* (**S**–**X**) probes. (**A**,**D**,**G**,**J**,**M**,**P**,**S**,**V**) *Runx2*^fl/+^, (**B**,**E**,**H**,**K**,**N**,**Q**,**T**,**W**) *Runx2*^fl/−^, and (**C**,**F**,**I**,**L**,**O**,**R**,**U**,**X**) *Runx2*^fl/−Cre^ mice. The boxed regions in (**A**–**C**), (**G**–**I**), (**M**–**O**), and (**S**–**U**) are magnified in (**D**–**F**), (**J**–**L**), (**P**–**R**), and (**V**–**X**). Scale bars = 200 μm. The number of mice analyzed was as follows: *Runx2*^fl/+^: 2, *Runx2*^fl/−^: 2, and *Runx2*^fl/−Cre^: 3. In situ hybridization using sense probes did not yield any significant signals.

**Figure 5:**
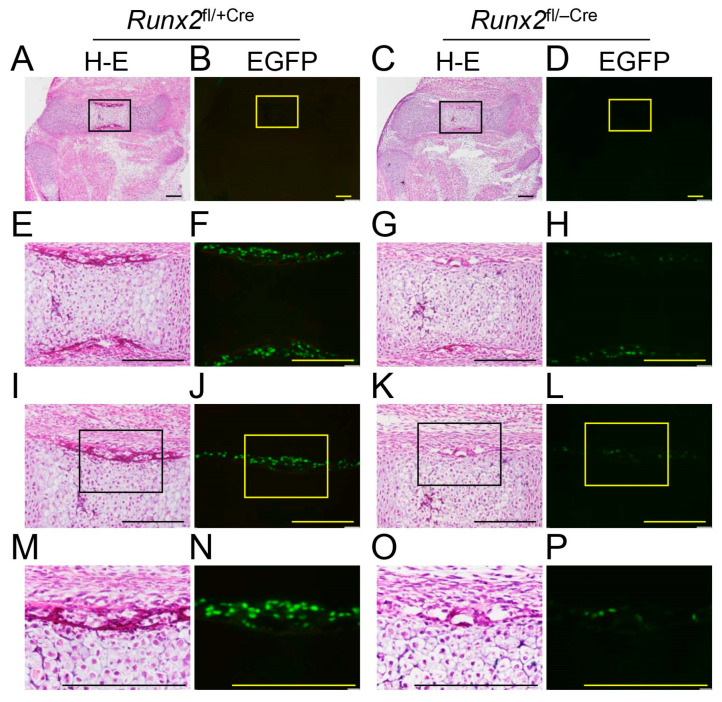
Comparison of osteoblast differentiation by 2.3 kb *Col1a1* EGFP-Cre expression in the bone collar of *Runx2*^fl/+Cre^ and *Runx2*^fl/−Cre^ mice at E15.5. (**A**–**P**) Frozen sections of the femurs. (**A**,**B**,**E**,**F**,**I**,**J**,**M**,**N**) *Runx2*^fl/+Cre^ and (**C**,**D**,**G**,**H**,**K**,**L**,**O**,**P**) *Runx2*^fl/−Cre^ mice. H&E staining was performed after observing the EGFP fluorescence signal. The boxed regions are magnified in the lower panels. Scale bars = 200 μm. The number of mice analyzed was as follows: *Runx2*^fl/+Cre^: 1 and *Runx2*^fl/−Cre:^ 1.

In situ hybridization was performed using *Col2a1*, *Col10a1*, *Spp1*, and *Col1a1* probes (Figure 4). *Col2a1* is expressed in resting and proliferating chondrocytes, *Col10a1* is expressed in hypertrophic chondrocytes, *Spp1* is expressed in terminal hypertrophic chondrocytes and immature osteoblasts, and *Col1a1* is weakly expressed in preosteoblasts and strongly expressed in mature osteoblasts [2]. *Col2a1* was similarly detected in resting and proliferating chondrocytes among *Runx2*^fl/+^, *Runx2*^fl/−^, and *Runx2*^fl/−Cre^ mice (Figure 4A–F). *Col10a1* expression was detected in the metaphyses of *Runx2*^fl/+^ and *Runx2*^fl/−^ mice, whereas it was detected in the diaphyses of *Runx2*^fl/−Cre^ mice (Figure 4G–L). *Spp1* was detected in the terminal hypertrophic chondrocytes in the diaphysis and osteoblasts in the bone collar in *Runx2*^fl/+^ and *Runx2*^fl/−^ mice, whereas it was weakly detected only in the bone collar in *Runx2*^fl/−Cre^ mice (Figure 4M–R). *Col1a1* was strongly detected in the bone collar of *Runx2*^fl/+^ and *Runx2*^fl/−^ mice but weakly detected in *Runx2*^fl/−Cre^ mice (Figure 4S–X). In accordance with these data, the number of EGFP-Cre-positive cells in the perichondrium of the femurs was much lower, and the intensity of EGFP-Cre was weaker in *Runx2*^fl/−Cre^ mice than in *Runx2*^fl/+Cre^ mice at E15.5 (Figure 5). These findings indicate that the terminal differentiation of chondrocytes and osteoblast differentiation in the bone collar are retarded in *Runx2*^fl/−Cre^ mice compared to those in *Runx2*^fl/−^ mice.

### 2.4. Expression of the Marker Genes for Terminal Hypertrophic Chondrocytes Was Reduced in Runx2^fl/−Cre^ Mice

**Figure 6:**
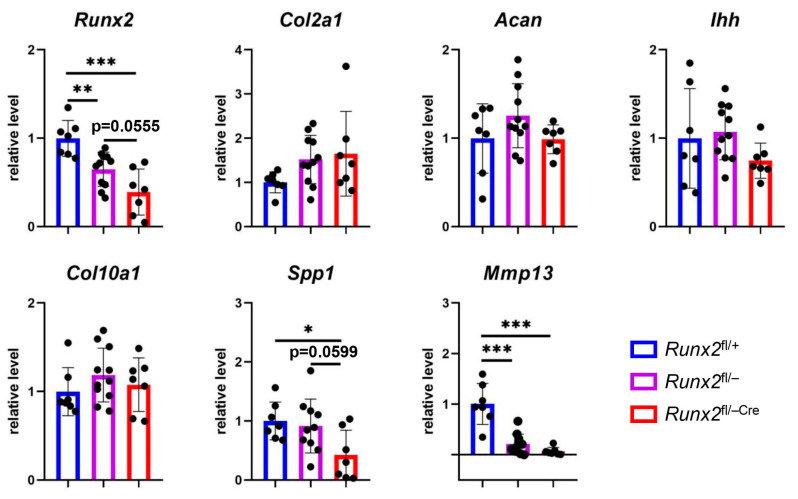
Real-time RT-PCR analysis of genes related to chondrocyte differentiation. RNA was extracted from the femurs and tibiae of *Runx2*^fl/+^, *Runx2*^fl/−^, and *Runx2*^fl/−Cre^ embryos at E15.5. The values in *Runx2*^fl/+^ mice were defined as 1, and the relative levels are shown. The number of mice analyzed was as follows: *Runx2*^fl/+^: 7, *Runx2*^fl/−^: 11, and *Runx2*^fl/−Cre^: 7. Data are the mean ± SD. * Versus *Runx2*^fl/+^ mice. * *p* < 0.05, ** *p* < 0.01, *** *p* < 0.001.

We examined the expression of chondrocyte marker genes using real-time reverse transcription (RT) polymerase chain reaction (PCR) using E15.5 femurs and tibiae (Figure 6). *Runx2* expression in *Runx2*^fl/−Cre^ mice was lower than that in *Runx2*^fl/+^ mice and marginally lower than that in *Runx2*^fl/−^ mice. The expression of *Col2a1* and *Acan*, which are expressed in resting and proliferating chondrocytes, was similar in *Runx2*^fl/+^, *Runx2*^fl/−^, and *Runx2*^fl/−Cre^ mice. The expression levels of *Ihh*, which is expressed in prehypertrophic chondrocytes, and *Col10a1* were comparable among *Runx2*^fl/+^, *Runx2*^fl/−^, and *Runx2*^fl/−Cre^ mice. *Spp1* expression in *Runx2*^fl/−Cre^ mice was significantly and marginally lower than that in *Runx2*^fl/+^ and *Runx2*^fl/−^ mice, respectively. The expression of *Mmp13*, which is mainly expressed in terminal hypertrophic chondrocytes [2], was significantly lower in *Runx2*^fl/−^ and *Runx2*^fl/−Cre^ mice than in *Runx2*^fl/+^ mice, with a greater reduction in *Runx2*^fl/−Cre^ mice.

### 2.5. RNA Sequence Analysis Showed That Nell1 Expression Is Downregulated in Runx2^fl/−Cre^ Mice Compared with Runx2^fl/−^ Mice More than Two-Fold

**Figure 7:**
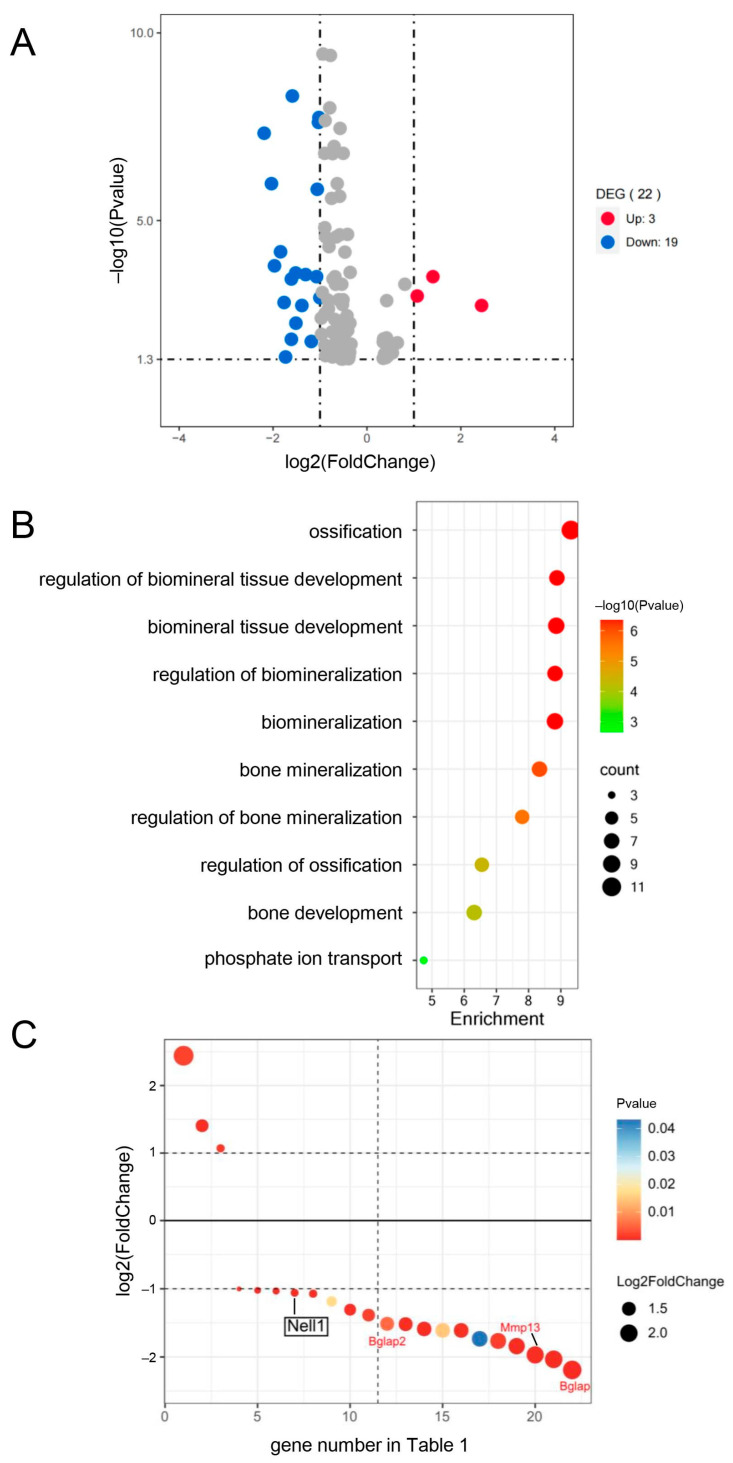
RNA-seq analyses of femurs and tibiae of *Runx2*^fl/−^ and *Runx2*^fl/−Cre^ embryos at E15.5. (**A**) Volcano plot analysis of 104 differentially expressed genes (DEGs) with a *p*-value < 0.05 in *Runx2*^fl/−Cre^ embryos compared with *Runx2*^fl/−^ embryos. X-axis: fold change (log2); Y-axis: *p*-value (–log10). Three red and nineteen blue points indicate significantly upregulated and downregulated genes (*p*-value <0.05, fold change >2), respectively. (**B**) GO term enrichment of 22 DEGs in biological processes. (**C**) The 22 DEGs are shown according to their numbers in Table 1. X-axis: number of DEGs in Table 1; Y-axis: fold change (log2). The dot size corresponds to the fold change (log2), and the color represents the *p*-value.

To investigate the reason for retarded terminal chondrocyte differentiation in *Runx2*^fl/−Cre^ mice, we compared the mRNA sequences extracted from the femurs and tibiae of *Runx2*^fl/−Cre^ and *Runx2*^fl/−^ mice at E15.5. One hundred and four genes were significantly changed (*p*-value < 0.05), three of which were upregulated, and nineteen were downregulated by more than two-fold (Figure 7A). Ossification and related GO terms in the biological process were enriched in the 22 genes, and 4 genes, including *Nell1*, *Bglap2*, *Mmp13*, and *Bglap*, were included in ossification (Figure 7B,C, Table 1). Although *Spp1* expression in *Runx2*^fl/−Cre^ mice was six times lower than that in *Runx2*^fl/−^ mice, it was not selected because the *p*-value was 0.06. EGFP-Cre was expressed only in the perichondrium of the femur and tibia at E15.5 (Figure 5). Therefore, deletion of Runx2 in the perichondrium should decelerate the terminal differentiation of chondrocytes in *Runx2*^fl/−Cre^ mice at E15.5. Thus, genes responsible for deceleration should be expressed in the perichondrium and encode the secreted proteins. Nell1 is a secreted protein that has been reported to be expressed in the perichondrium and chondrocytes and to induce chondrocyte maturation [15,16,18]. Moreover, *Nell1* expression has been reported to be regulated by Runx2 [16,19,20].

### 2.6. Nell1 Was Downregulated in the Perichondrium of Runx2^fl/−Cre^ Mice, Overexpression of Runx2 Induced Nell1 Expression, and Runx2 siRNA Reduced Nell1 Expression

**Figure 8:**
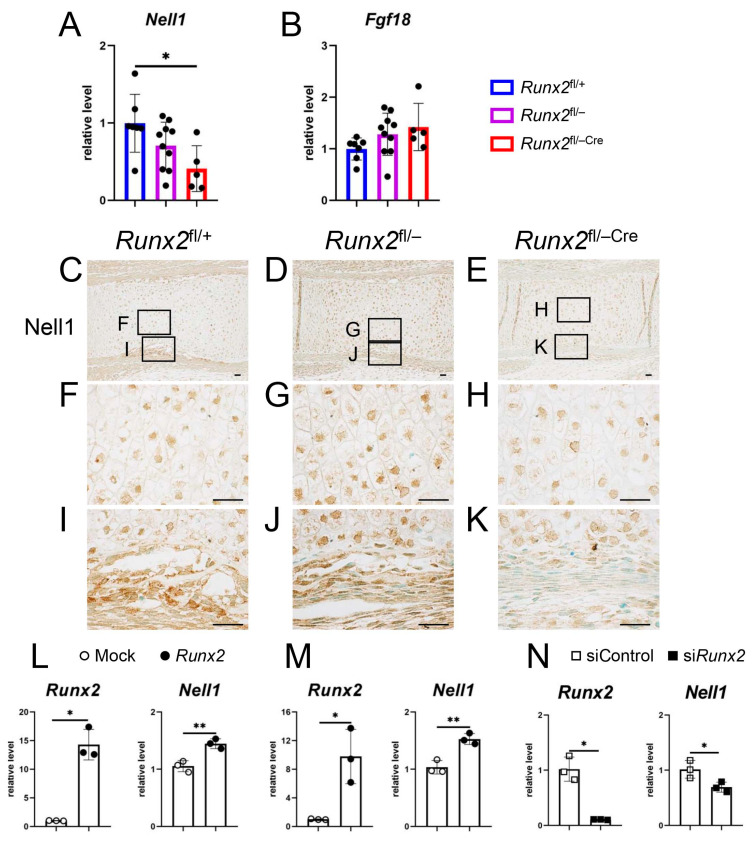
Nell1 expression in the femurs and tibiae of *Runx2*^fl/+^, *Runx2*^fl/−^, and *Runx2*^fl/−Cre^ embryos at E15.5. (**A**,**B**) Real-time RT-PCR analysis of *Nell1* (**A**) and *Fgf18* (**B**) expression using RNA from femurs and tibiae at E15.5. The values in *Runx2*^fl/+^ mice were defined as 1, and relative levels are shown. The number of mice analyzed was as follows: *Runx2*^fl/+^: 7, *Runx2*^fl/−^: 10, and *Runx2*^fl/−Cre^: 5. Data are the mean ± SD. * Versus *Runx2*^fl/+^ mice. * *p* < 0.05. (**C**–**K**) Immunohistochemical analysis of femoral sections in *Runx2*^fl/+^ (**C**,**F**,**I**), *Runx2*^fl/−^ (**D**,**G**,**J**), and *Runx2*^fl/−Cre^ (**E**,**H**,**K**) embryos using an anti-Nell1 antibody. The boxed regions in (**C**–**E**) are magnified in (**F**,**I**), (**G**,**J**), and (**H**,**K**), respectively. Scale bars: 100 μm. The number of mice analyzed was as follows: *Runx2*^fl/+^: 2, *Runx2*^fl/−^: 2, and *Runx2*^fl/−Cre^: 1. (**L**,**M**) Real-time RT-PCR analysis of *Runx2* and *Nell1* by *Runx2* overexpression. Primary osteoblasts were transfected with control (Mock) or *Runx2*-expressing vector, and RNA was extracted 24 h (**L**) and 48 h (**M**) after transfection. (**N**) Real-time RT-PCR analysis of *Runx2* and *Nell1* by *Runx2* siRNA. Primary osteoblasts were transfected with siRNA for control (siControl) or *Runx2* (si*Runx2*), and RNA was extracted 24 h after transfection. The values in the Mock or siControl groups were defined as 1, and the relative levels are shown. n = 3. Versus Mock, * *p* < 0.05, ** *p* < 0.01. Three and two independent experiments for *Runx2* overexpression and si*Runx2*, respectively, were performed, and representative data are shown.

Real-time RT-PCR using RNA from femurs and tibiae at E15.5 showed that the expression of *Nell1* in *Runx2*^fl/−Cre^ mice was significantly lower than that in *Runx2*^fl/+^ mice, although the reduction was not significant compared to that in *Runx2*^fl/−^ mice (Figure 8A). Although Fgf18 in the perichondrium had been shown to regulate chondrocyte maturation [9], *Fgf18* expression was comparable among *Runx2*^fl/+^, *Runx2*^fl/−^, and *Runx2*^fl/−Cre^ mice (Figure 8B). The expression of Nell1 was also examined by immunohistochemistry using an Nell1 antibody. Nell1 was detected similarly in chondrocytes among *Runx2*^fl/+^, *Runx2*^fl/−^, and *Runx2*^fl/−Cre^ mice, but Nell1 expression in the perichondrium in *Runx2*^fl/−Cre^ mice was reduced compared to that in *Runx2*^fl/+^ and *Runx2*^fl/−^ mice (Figure 8C–K). In accordance with previous reports [16,19,20], *Runx2* overexpression in primary osteoblasts induced *Nell1* expression (Figure 8L,M). Moreover, *Runx2* siRNA reduced *Nell1* expression in primary osteoblasts (Figure 8N).

### 2.7. Both Trabecular and Cortical Bone Were Severely Reduced in Runx2^fl/−Cre^ Femurs at 11 Weeks of Age

**Figure 9:**
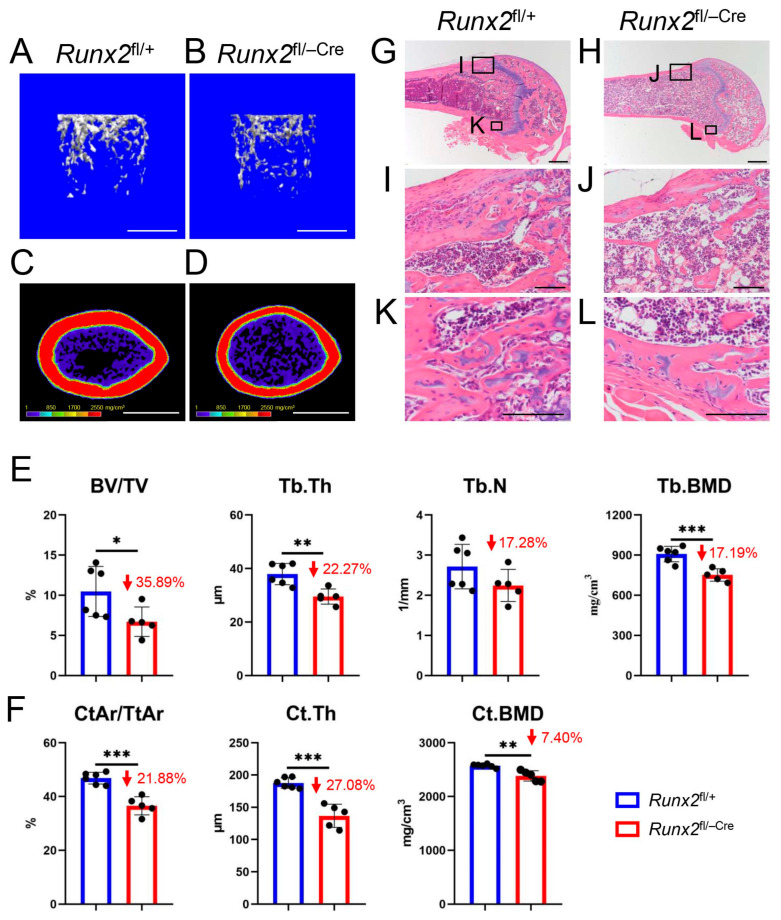
Micro-CT and histological analyses of male *Runx2*^fl/+^ and *Runx2*^fl/−Cre^ mice at 11 weeks of age. (**A**–**D**) Three-dimensional trabecular bone architecture of the distal femoral metaphysis (**A**,**B**) and micro-CT images of the cortical bone at mid-diaphysis (**C**,**D**) in the femurs of *Runx2*^fl/+^ (**A**,**C**) and *Runx2*^fl/−Cre^ (**B**,**D**) mice. Scale bars = 1 mm. (**E**) BV/TV, Tb.Th, Tb.N, and Tb.BMD in the trabecular bone. (**F**) CtAr/TtAr, Ct.Th, and Ct.BMD in the cortical bone. The number of mice analyzed was as follows: *Runx2*^fl/+^: 6 and *Runx2*^fl/−Cre^: 5. Data are the mean ± SD. * Versus *Runx2*^fl/+^ mice. * *p* < 0.05, ** *p* < 0.01, *** *p* < 0.001. (**G**–**L**) H&E staining of femoral sections from *Runx2*^fl/+^ and *Runx2*^fl/−Cre^ mice. The boxed regions in (**G**,**H**) are magnified in (**I**,**K**), and (**J**,**L**), respectively. Scale bars = 0.5 mm in (**G**,**H**); scale bars = 100 μm in (**I**–**L**). The number of mice analyzed was as follows: *Runx2*^fl/+Cre^: 1 and *Runx2*^fl/−Cre^: 1.

Micro-CT analysis showed that the trabecular parameters, including bone volume/total volume (BV/TV), trabecular thickness (Tb.Th), trabecular number (Tb.N), and trabecular bone mineral density (Tb.BMD), and the cortical parameters, including cortical area/total area (CtAr/TtAr), cortical thickness (Ct.Th), and cortical BMD (Ct.BMD), were severely reduced in *Runx2*^fl/−Cre^ femurs compared to those in *Runx2*^fl/+^ femurs (Figure 9A–F). The reduction compared to that in wild-type mice was more severe in *Runx2*^fl/−Cre^ mice than in *Runx2*^+/−^ mice [22]. Histological analysis also showed a reduction in trabecular and cortical bones in *Runx2*^fl/−Cre^ mice compared to that in *Runx2*^fl/+^ mice (Figure 9G–L). These findings indicate the requirement of Runx2 in committed osteoblasts in adult mice.

## 3. Discussion

Deletion of *Runx2* by *Col1a1* Cre resulted in retarded terminal differentiation of the chondrocytes. Nell1 expression was reduced in the perichondrium of *Runx2*^fl/−Cre^ mice, and overexpression and knockdown of *Runx2* induced and reduced *Nell1* expression, respectively. These findings, combined with previous reports, indicate that Runx2 in the perichondrium positively regulates the terminal differentiation of chondrocytes by inducing *Nell1* expression.

Removal of the perichondrium from chick tibiotarsi elongates the tibiotarsi and increases the layer of proliferating chondrocytes, followed by an increase in the *Col10a1* expressing region during organ culture, suggesting that the perichondrium inhibits chondrocyte proliferation and maturation [8]. However, the growth of perichondrium-removed chick tibiotarsi was similar to that of perichondrium-intact tibiotarsi when cultured in the chorioallantoic membrane [23]. Thus, the function of the perichondrium against chondrocytes remains controversial. In contrast to our findings, Runx2 in the perichondrium of long bones was shown to negatively regulate chondrocyte proliferation and maturation by inducing *Fgf18*, which is expressed in the perichondrium [9]. Twist1, which is expressed in the perichondrium, inhibits Runx2 function, and *Twist1* mutant mice were shown to inhibit chondrocyte maturation, which was interpreted in terms of the shortening of the *Col10a1*-expressing layer [9]. However, the phenotypes of *Twist1* mutant mice showed that the reduced *Col10a1* layer was caused by enhanced chondrocyte maturation and endochondral ossification. Thus, the controversy was caused by the misinterpretation of the phenotypes of *Twist1* mutant mice, and the correct interpretation of the phenotype indicates that Runx2 in the perichondrium enhances chondrocyte maturation. Furthermore, Fgf18 enhances the terminal differentiation of chondrocytes [10,11]. Thus, it is still possible that Runx2 in the perichondrium enhances chondrocyte maturation through *Fgf18* induction. However, *Fgf18* expression was similar in *Runx2*^fl/+^, *Runx2*^fl/−^, and *Runx2*^fl/−Cre^ mice.

Nell1 and Runx2 have similar expression patterns in the calvaria and long bones, and *Nell1* expression is severely reduced in *Runx2*^−/−^ mice [15,16,19]. In long bones, both are strongly expressed in prehypertrophic, hypertrophic, and terminal hypertrophic chondrocytes, perichondrium, and osteoblasts in the bone marrow and bone collar [15,16]. Both Runx2 and Nell1 enhance chondrocyte maturation and osteoblast differentiation, and *Nell1* expression is directly regulated by Runx2 [3,13,16,18,19,20]. We also confirmed that *Nell1* expression is regulated by Runx2 (Figure 8L–N). *Col10a1*-expressing hypertrophic chondrocytes were reduced in chondrocyte-specific *Nell1*-deficient mice [18], whereas maturation to *Col10a1*-positive hypertrophic chondrocytes was not impaired in *Runx2*^fl/−Cre^ mice. Therefore, Nell1 in chondrocytes may be sufficient for maturation into *Col10a1* expressing hypertrophic chondrocytes. As the differentiation of *Col10a1*-expressing hypertrophic chondrocytes to *Spp1* expressing terminal hypertrophic chondrocytes was retarded in *Runx2*^fl/−Cre^ mice, Nell1 in the perichondrium, in addition to that in chondrocytes, is likely to be necessary for normal terminal differentiation.

## 4. Materials and Methods

### 4.1. Mice

*Runx2* flox mice were generated by homologous recombination using a targeting vector containing Loxps upstream and downstream of exon 2, as previously described [2]. *Runx2*^+/−^ mice were generated as previously described [22], and 2.3 kb *Col1a1* EGFP-Cre transgenic mice, which produce EGFP-Cre fusion protein specifically in osteoblasts, were generated using the 2.3 kb *Col1a1* promoter and EGFP-Cre DNA, as previously described [21]. *Runx2*^fl/−^ and *Runx2*^fl/−Cre^ mice were generated by crossing *Runx2*^fl/+Cre^ with *Runx2*^+/−^ mice. *Runx2*^fl/−Cre^ mice express *Runx2* in one allele and in chondrocytes but not in osteoblasts. *Runx2*^fl/+^ mice were generated and maintained on a C57BL/6N background; 2.3-kb *Col1a1* EGFP-Cre transgenic mice were generated on a B6C3H F1 background and backcrossed with C57BL/6N mice more than eight times; and *Runx2*^+/^^−^ mice were generated on a 129Ola/C57BL6 background and backcrossed with C57BL/6N at least 12 times. Before the study, all experimental protocols were reviewed and approved by the Animal Care and Use Committee of the Nagasaki University Graduate School of Biomedical Sciences (No. 2401311920-4). Animals were housed three per cage in a pathogen-free environment on a 12 h light cycle at 22 ± 2 °C, with standard chow (CLEA Japan, Tokyo, Japan) and free access to tap water. All relevant guidelines for working with animals were followed.

### 4.2. Skeletal Staining and Micro-CT Analysis

The vertebral, thoracic cage, and upper limb bones were stained with alcian blue and alizarin red, as described previously [22]. Micro-CT analysis was performed using a micro-CT system (R_mCT; Rigaku Corporation, Tokyo, Japan). Data from the scanned slices were used for three-dimensional analysis to calculate the femoral morphometric parameters. Trabecular bone parameters were measured in the distal femoral metaphysis of the femur. Craniocaudal scans of approximately 2.4 mm (0.5 mm from the growth plate) for 200 slices in 12 μm increments were obtained. The cortical bone parameters were measured at the mid-diaphysis of the femurs. The threshold for the mineral density was set at 500 mg/cm^3^.

### 4.3. Histological Analyses

Mice were fixed in 4% paraformaldehyde/0.1 M phosphate buffer, E15.5 embryos were undecalcified, and other stages of mice were decalcified and embedded in paraffin. Sections (4 μm thick) were stained with hematoxylin and eosin (H&E), von Kossa, or tartrate-resistant acid phosphatase (TRAP). In situ hybridization was conducted using mouse *Col2a1*, *Col10a1*, *Spp1*, and *Col1a1* antisense and sense probes, as described previously [2]. The sections were then counterstained with methyl green. To observe osteoblast differentiation in the bone collars of femurs, *Runx2*^fl/+Cre^ and *Runx2*^fl/−Cre^ embryos at E15.5 were fixed with 4% paraformaldehyde at 4 °C for 2 h, washed with PBS at 4 °C for 1 h, immersed in 20% sucrose at 4 °C overnight, and embedded in O. C. T. compound (Sakura Finetek, Tokyo, Japan), frozen in a refrigerated installation (Rikakikai UT-2000F, Tokyo, Japan) containing −100 °C hexane and pentane (10:3), and sectioned at 7 μm thickness using a Leica CM3050S (Leica Biosystems, Tokyo, Japan). The same section was first subjected to fluorescence imaging, followed by H&E staining. Immunohistochemistry was performed using a polyclonal sheep anti-Nell1 antibody (R&D Systems, Catalog #AF7109, Minneapolis, MN, USA) and a polyclonal donkey anti-sheep IgG horseradish peroxidase-conjugated antibody (R&D Systems, Catalog #HAF016).

### 4.4. Real-Time Reverse Transcription (RT)-PCR

Total RNA was extracted using ISOGEN (Wako, Osaka, Japan). For cDNA synthesis, 500 ng of total RNA was reverse-transcribed using ReverTra Ace qPCR Master with gDNA Remover (Toyobo, Osaka, Japan). Real-time RT-PCR was performed using the THUNDERBIRD SYBR qPCR Mix (Toyobo) and a Light Cycler 480 real-time PCR system (Roche Diagnostics, Tokyo, Japan). The values were normalized to that of *Actb*. Primer sequences are shown in Supplementary Table S1. The expression of *Col10a1* was examined using TaqMan probes Mm00487041_m1 for *Col10a1* and Mm02619580_g1 for *Actb* (Thermo Fisher Scientific, Tokyo, Japan).

### 4.5. RNA Sequencing Analysis

Total RNA was prepared from the femurs and tibiae, in which skin and muscle had been removed, of three *Runx2*^fl/−^ and *Runx2*^fl/−Cre^ embryos at E15.5 using ISOGEN (Wako). RNA sequencing was performed on the DNBSEQ platform (MGI Tech, Shenzhen, China) by Tokai Biotechnology Co., Ltd. (Osaka, Japan). Sequence quality was first surveyed with FastQC (version 0.12.1). Paired-end raw reads were trimmed using Trimmomatic and mapped to the mouse genome assembly mm10. The GRCm38 reference genome was obtained from the UCSC Genome Browser database using Bowtie2 (version 2.7.11b). mRNA expression was quantified using featureCounts (version 2.0.3). Differentially expressed mRNA was calculated using the DESeq2 R package (version 1.38.3) with a cutoff fold change >2 and FDR-adjusted *p*-value (*p*-value) < 0.05. The GO enrichment analysis was performed using the function “enrichGO” from the R package clusterProfiler (version 4.6.2).

### 4.6. Cell Culture and Runx2 Overexpression and Knockdown

Primary osteoblasts were isolated from the calvariae of newborn wild-type mice by sequential digestion with 0.1% collagenase A and 0.2% dispase (Sigma, St. Louis, MO, USA) for 10 min at 37 °C. Osteoblastic cells from the third to fifth fractions were pooled and plated on 48-well plates at a density of 4.0 × 10^4^ cells/cm^2^ in alpha-modified Eagle’s Minimum Essential Medium (αMEM; Merck, Darmstadt, Germany) supplemented with 10% fetal bovine serum (Nichirei, Tokyo, Japan), L-glutamine (Merck), and 100 U/mL penicillin (Nacalai Tesque, Inc., Kyoto, Japan) and 100 μg/mL streptomycin (Nacalai Tesque). Cells were transfected with 0.1 μg of control (pSG5-Mock) or *Runx2*-expressing (pSG5-*Runx2*) vectors using X-tremeGENE9 (Roche Diagnostics). Cells were also transfected with 90 pmol *Runx2* siRNA (triplex; Thermo Scientific) or scrambled RNA using X-tremeGENE360 (Roche Diagnostics). The transfected cells were cultured for 24 h or 48 h.

### 4.7. Statistical Analysis

Values are expressed as the mean ± SD. Statistical analyses of two groups were performed by the Student *t*-test, and those of more than three groups were conducted by one-way ANOVA by GraphPad Prism (8.0.1). Statistical significance was set at *p* < 0.05.

### 4.8. Graphical Abstract

The graphical abstract was drawn using FigDraw 2.0.

## 5. Conclusions

In conclusion, contrary to a previous report [9], Runx2 in the perichondrium positively regulates chondrocyte terminal differentiation. Therefore, in endochondral ossification, Runx2 induces chondrocyte maturation directly and indirectly through the perichondrium by inducing Nell1 expression. This report added a novel function of Runx2 in endochondral ossification.

## Figures and Tables

**Table 1:** List of 22 DEGs with GO terms and expression in *Runx2*^fl/−^ and *Runx2*^fl/−Cre^ embryos at E15.5.

No.	Genes	GO Terms (Biological Process)	*Runx2* ^fl/^^−^			*Runx2* ^fl/^^−^^Cre^		
			1	2	3	1	2	3
1	*Nek10*	cilium assembly	10	4	14	34	64	52
2	*Neto1*	synaptic signaling	48	71	52	178	157	108
3	*Grip2*	synapse organization	85	128	129	263	188	256
4	*Bcan*	nervous system development	216	183	183	98	88	99
5	*Lratd1*	lipid metabolic process	833	812	675	328	451	336
6	*Enpp6*	small molecule metabolic process	1425	1164	1100	475	730	558
7	*Nell1*	bone mineralization	481	522	425	168	258	244
8	*Pacsin1*	endocytosis	440	335	333	119	221	175
9	*Steap4*	transition metal ion homeostasis	4607	3578	3449	1197	2212	1584
10	*Cpa3*	regulation of blood pressure	153	215	151	51	72	82
11	*Cdkn2b*	regulation of cell population proliferation	145	124	142	32	72	50
12	*Bglap2*	bone mineralization	142	123	127	22	74	38
13	*Hp*	acute-phase response	217	129	214	46	91	55
14	*Hapln4*	extracellular matrix organization	218	213	195	62	62	80
15	*Gabrb2*	chemical synaptic transmission	132	82	119	12	49	46
16	*Lipc*	lipoprotein metabolic process	176	107	111	34	59	33
17	*Gvin2*	regulation of cell growth	61	44	74	7	23	23
18	*Sstr2*	regulation of gastrointestinal motility	106	86	72	38	24	14
19	*Xdh*	purine nucleoside catabolic process	156	95	182	26	58	35
20	*Mmp13*	bone mineralization	21,789	11,610	16,420	2993	5610	3856
21	*Muc5b*	epithelial cell differentiation	153	95	188	27	44	34
22	*Bglap*	bone mineralization	250	196	150	22	69	36

The mRNA expression levels of the indicated genes in three embryos of each genotype are shown in the right column.

## Data Availability

The original contributions presented in this study are included in the article/Supplementary Materials. Further inquiries can be directed to the corresponding author.

## Supplementary Materials

The following supporting information can be downloaded at: https://www.mdpi.com/article/10.3390/ijms27031266/s1.
