# Supplementary material for: Runx2 in the Perichondrial Osteoblasts Enhances Terminal Differentiation of Chondrocytes Through Nell1 Induction
Source: Int J Mol Sci. 2026 Jan 27;27(3):1266. doi: 10.3390/ijms27031266 (PMC12898123; doi:10.3390/ijms27031266)
Supplement: Supplementary file 1 [file ijms-27-01266-s001.zip › ijms-4102564-supplementary.pdf]

Supplementary Fig.S1

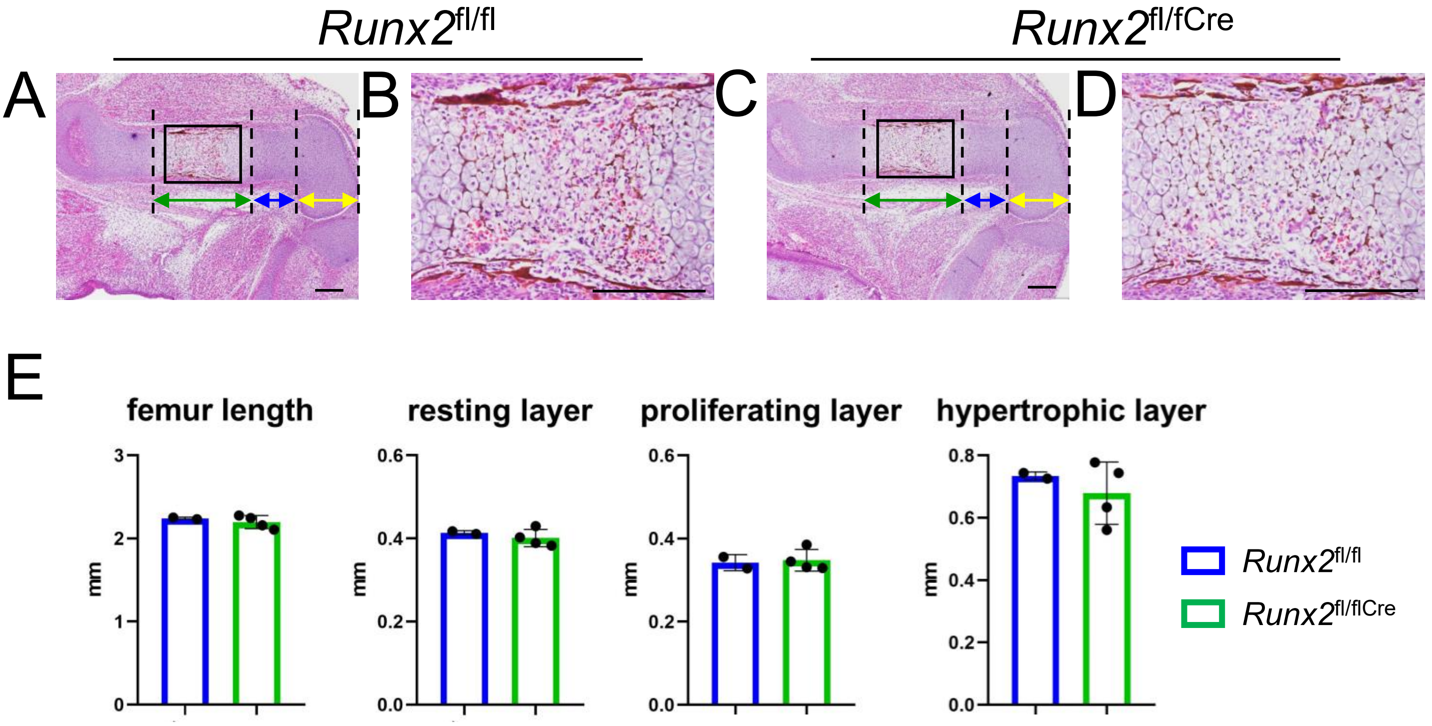

Supplementary Fig. S1

Histological analysis of *Runx2*$^{fl/fl}$ and *Runx2*$^{fl/flCre}$ embryos at E15.5. (A–D) H-E staining of femoral sections from *Runx2*$^{fl/fl}$(A, B) and *Runx2*$^{fl/flCre}$(C, D) embryos. The boxed regions in A and C are magnified in B and D. Scale bars=200 μm. (E) Lengths of femurs and layers of resting (yellow arrows), proliferating (blue arrows), and hypertrophic (green arrows) chondrocytes. The number of mice analyzed: *Runx2*$^{fl/fl}$: 2 and *Runx2*$^{fl/flCre}$: 4.

Supplementary Table S1

Primer sequences for real-time RT-PCR :

	forward	reverse
	5'-----------------------------------3'	5'-------------------------------------3'
Actb	CCACCCGCGAGCACAGCTTC	TTGTCGACGACCAGCGCAGC
Runx2	AACAAGACCCTGCCCGTG	TGAAACTCTTGCCTCGTCCG
Col2a1	ATCTGGTTTGGAGAGACCAT	CTCTACATCATTGGAGCCCT
Acan	ACCCGGTACCCTACAGAGAC	GTCCACCCCTCCTCACATTG
Ihh	TTCAAGGACGAGGAGAACACG	TTCAGACGGTCCTTGCAGC
Spp1	GCAGAATCTCCTTGCGCCAC	CGAGTCCACAGAATCCTCGC
Mmp13	CTTCTGGCACACGCTTTTCC	ATGGGAAACATCAGGGCTCC
Nell1	TCCTGGGTAGATGGTGACAA	CATTGGCCAGAAATATGCAC
Fgf18	ACAGATACCTTCGGGAGTCA	TCTTGGTGAAGCCCACATAC
